# Biopreservative Effect of the Tunisian Halophyte *Lobularia maritima* Flavonoid Fraction, Used Alone and in Combination with Linalool in Stored Minced Beef Meat

**DOI:** 10.3390/metabo13030371

**Published:** 2023-03-02

**Authors:** Boutheina Ben Akacha, Stefania Garzoli, Rania Ben Saad, Faical Brini, Wissem Mnif, Miroslava Kačániová, Anis Ben Hsouna

**Affiliations:** 1 Laboratory of Biotechnology and Plant Improvement, Centre of Biotechnology of Sfax, University of Sfax, Sfax 3018, Tunisia; 2 Department of Chemistry and Technologies of Drug, Sapienza University, 00185 Rome, Italy; 3 Department of Chemistry, College of Sciences, University of Bisha, P.O. Box 199, Bisha 61922, Saudi Arabia; 4 Institute of Horticulture, Faculty of Horticulture, Slovak University of Agriculture, Tr. A. Hlinku 2, 949 76 Nitra, Slovakia; 5 Department of Bioenergy, Food Technology and Microbiology, Institute of Food Technology and Nutrition, University of Rzeszow, 4 Zelwerowicza St., 35601 Rzeszow, Poland; 6 Department of Environmental Sciences and Nutrition, Higher Institute of Applied Sciences and Technology of Mahdia, University of Monastir-Tunisia, Monastir 5000, Tunisia

**Keywords:** chemical composition, flavonoid extract, linalool, antioxidant activity, synergistic effect, minced beef meat, microbial spoilage, multivariate analysis

## Abstract

In the present study, *Lobularia maritima* (*Lm*) flavonoid extract (*Lm*FV) was characterized by HPLC analyses and five compounds were detected. Further, to describe the chemical content of the matrix, GC-MS analyses after silylation were performed; the obtained results showed the presence of a large number of components belonging to several chemical classes, mostly sugar alcohols, sugars, fatty acids, and terpenes. Firstly, the antibacterial activities of this fraction and linalool (Lin) were evaluated against eight foodborne pathogenic strains with MIC values between 2.3 and 5.8 mg/mL and 0.23 and 0.7 mg/mL, respectively. Then, the antioxidant activity of both was evaluated by the DPPH antiradical test and the phosphomolybdenum test. Furthermore, the biopreservative effect of *Lm*FV alone and in combination with Lin on minced beef stored at 4 °C for 14 days was evaluated using microbiological and physiochemical tests. *Lm*FV at 4.6% alone significantly reduced microbial spoilage in ground meat (*p* < 0.05). The combination of *Lm*FV (4.6%) and Lin (0.46%) was more effective than *Lm*FV alone in inhibiting bacterial contamination, reducing TBARS values and the risk of bacterial contamination, and reducing the accumulation of Met myoglobin (MetMb). This combination, therefore, extends the shelf life of the product by about 10 days. Based on these microbiological results and physicochemical parameters, it can be stated that the addition of Lin potentiates the flavonoid fraction of *L. maritima* more strongly against the deterioration of meat quality by significantly improving its biopreservative effect as a natural conservative.

## 1. Introduction

The name “flavonoids” comes from the term “flavado,” which refers to the outer layer of orange peel [1]. Flavonoids are the most abundant group of phenolics in terms of structural diversity. Nine thousand have been confirmed so far. These molecules are involved in the coloring of flowers, fruits, and leaves and are thought to protect tissues from the harmful effects of UV radiation [2].

Flavonoids can be present in all parts of plants [3,4]. The majority of flavonodes in plants are glycosylated because glycosylation makes them less reactive and more water-soluble, allowing them to be stored in the vacuoles of the epidermal cells of flowers, the epidermis and mesophyll of leaves, and the parenchyma of stems and roots [5]. A class of polyphenols is classified as flavonoids [6,7]. According to where the aromatic ring is located in the benzopyran portion, they can be divided into four major classes. There are several subclasses of flavonoids of plant origin, including flavonol, flavone, flavanone, flavanol, isoflavone, and anthocyanin [8]. The phenylbenzopyran ring is linked to a C_15_ (C_6_-C_3_-C_6_) backbone in the chemical structure of flavonoids. In vitro and in vivo research has shown that the flavonoids from plants have anticancer, antioxidant, and anti-inflammatory properties [3,5,9], and they have been proven to have very high antibacterial activity against a variety of microorganisms, including bacteria [8,9,10,11] and fungi [12,13].

This makes these bioactive compounds a resource for preserving foods and decreasing the contamination of food-borne pathogens that have been causing significant public health problems worldwide.

According to scientific studies, halophytes high in antioxidants, such as polyphenols, flavonoids, and vitamins, are much more resistant to salt stress [14,15]. Previous research has shown that salt treatment (200 mM NaCl) increased the contents of phenolic compounds and flavonoids in maritime leaves, whereas higher salinity (400 mM NaCl) decreased the contents of these compounds. These findings were related to antioxidant activity. The leaf extract of *L. maritime* demonstrated greater DPPH free radical quenching activity at 200 mM NaCl than at 400 mM NaCl [15,16]. 

The nutritional value of meat is very high due to the presence of high-quality proteins, vitamins, minerals, and fats [17,18,19]. The oxidation process is the primary non-microbial cause of the degradation of these foods, particularly the oxidation of lipids rich in polyunsaturated fatty acids, which raises concerns about the quality and shelf life of meat and meat products. Natural antioxidants, such as flavonoids from halophytes, present a solution for improving the antioxidant capacity of meat, such that the overall quality and acceptability of meat and meat products can be improved.

Lin is also attractive in the food industry. The incorporation of Lin as a biopreservative ingredient allows for the inhibition of a wider range of microorganisms, including certain food-spoilage bacteria and foodborne pathogens, as it has been shown to have an exceptional inhibitory effect on Gram-positive and Gram-negative bacteria [20,21,22]. Besides its antibacterial activity, Lin is characterized by its powerful antioxidant activity against oxidative stress and oxidation processes [22,23,24], which makes it an excellent biopreservative to improve the microbial stability and safety of fresh meat in cold storage.

This work aimed to extract the flavonoid fraction of *L. maritima*, to describe its composition, and to determine its antioxidant and antibacterial activities against a large selection of foodborne pathogens.

To improve the biopreservative effect of *Lm*FV in minced beef meat, we tested it in combination with the bioactive component Lin. Thus, in the present work, the effect of *Lm*FV alone and in combination with Lin on the shelf life and microbiological and physicochemical properties of raw minced beef meat stored at 4 °C was evaluated.

## 2. Materials and Methods

### 2.1. Chemicals

Anhydrous sodium sulphate, linalool ((±)-3,7-dimethyl-1,6-octadien-3-ol), butylatedhydroxytoluene (BHT), streptomycin, thiazolyl blue tetrazolium bromide (MTT), and potassium phosphate buffer (K_3_PO_4_) were purchased from Sigma-Aldrich Co. (St. Louis, MO, USA). Muller–Hinton agar was purchased from Bio-Rad, Marnes-la-Coquette, France. Agar plates of MH agar and red-violet bile glucose medium were obtained from Oxoid Ltd., Basingstoke, UK.

For extraction and derivatization, ethanol, pyridine, and bis-(trimethylsilyl) tri-fluoroacetamide (BSTFA) were purchased from Sigma-Aldrich (Steinheim, Germany).

### 2.2. Preparation of Flavonoid Extracts (LmFV)

Flavonoids were extracted from *L. maritima* using the method described by Kao et al. [25], with minor physicochemical modifications. Briefly, 1 g of *L. maritima* powder was mixed with 30 mL of EtOH (80%), and the extract obtained was filtered and centrifuged for 30 min (5000 rpm at 4 °C), followed by solubilization of the filtrate obtained in 5 mL of deionized water and 5 mL of diluted H_2_SO_4_ (pH 2.0).

The crude extract of *L. maritima* was then purified using an SPE column. In this process, 1 mL of acidified crude extract of *L. maritima* was poured into the SPE cartridge before pre-activating the SPE cartridge with methanol and acidified water. The obtained flavonoids were eluted with 99% methanol and water, respectively. The methanol fractions were then vacuum-dried and dissolved in DMSO/EtOH.

Finally, the samples were filtered through a 0.22 μm membrane filter, and the purified fractions were used for HPLC-DAD analysis.

### 2.3. HPLC Analyses of LmFV

Regarding the HPLC instrumentation and separation conditions, a chromatography study was performed using HPLC-DAD (Agilent Technologies 1100 series, Fremont, CA, USA). Briefly, 10 µL of sample was injected using an HPLC system equipped with a G1315A autosampler and then separated on an Agilent Zorbax SB-C18 column (5 µm particle size, 4.6 × 150 mm; CA, USA) maintained at 38 °C. The mobile phase A was acetonitrile (HPLC-grade), and mobile phase B was 0.5% glacial acetic acid aqueous solution. The mobile phase gradient was: 0 min, 12% A; 0~20 min, 25% A; 20~35 min, 45% A; 35~40 min, 100 % A. The flow rate was 1.0 mL/min, and the column temperature was 35 °C. After 10 μL test solution was injected, the chromatographic peak area was obtained at a detection wavelength of 285 nm. The compounds in *L. maritima* were quantified according to the chromatographic peak area of standard substance. Results are expressed as µg/g of dry weight (DW).

### 2.4. GC-MS Analysis of the Extract after Derivatization

To describe the chemical content of the extract, a derivatization reaction was performed. For this purpose, 0.8 g of *L. maritima* powder was extracted with 250 mL of EtOH (96%). The extract was filtered and dried under reduced pressure at 40 °C to obtain 1.8 mg of residue.

Subsequently, 1 mg of extract was added to 300 µL of pyridine and 100 µL bis-(trimethylsilyl) tri-fluoroacetamide (BSTFA), with heating at 70 °C for 40 min. One microliter of the silylated sample was manually injected at 270 °C into the GC injector in the splitless mode.

The analysis was performed using a Clarus 500 model Perkin Elmer (Waltham, MA, USA) gas chromatograph coupled with a single quadrupole mass spectrometer (Clarus 500 model Perkin Elmer) equipped with FID (flame detector ionization) [26]. The capillary column was a Varian Factor Four (VF-1). The oven temperature program was as follows: 70 °C, then a gradient of 6 °C/min to 170 °C for 1.0 min, and a gradient of 8 °C/min to 250 °C for 35 min. Mass spectra were acquired in an electron impact mode. The identification of compounds was based on the percentage of similarity plus comparison of mass spectra (MS) using the NIST software data library, with the percentage of total ion chromatograms (TIC %). Relative percentages for quantification of the components were calculated by electronic integration of the GC-FID peak areas without using internal standard and factors correction.

### 2.5. Antioxidant Activity

#### 2.5.1. Assay of DPPH Radical Scavenging Activity

Antioxidant activity was measured using the 1,1-diphenyl-2-picryl-hydrazyl (DPPH) radical reduction assay (Sigma-Aldrich), and the free radical scavenging activity of *Lm*FV and Lin was determined according to the method of Ben Hsouna et al. [27], with some modifications. Aliquots of 2 mL of tested samples in ethanol were mixed with 2 mL of DPPH solution. The scavenging capacity was measured by spectrophotometry following the decrease in absorbance at 517 nm. A lower absorbance of the reaction mixture indicates a higher free radical scavenging activity. BHT (butylatedhydroxytoluene) was used as a standard. Radical scavenging activities, expressed as percent DPPH inhibition, were obtained using the following equation:

DPPH radical scavenging activity (%) = [(blank OD − sample OD)/blank OD] × 100(1)

The blank OD is the control reaction containing all reagents except the test compound. The sample OD is the absorbance of the test compound. The concentration of the extract allowing 50% inhibition (IC_50_) was calculated from the graph representing the percentage of inhibition as a function of extract concentration. The tests were performed in triplicate.

#### 2.5.2. Phosphomolybdenum Method

The total antioxidant activity of *Lm*FV was determined by Prieto et al. [28]. Briefly, 1 mL of *Lm*FV or Lin solutions at different concentrations was mixed with 1 mL of standard reagent solution (0.6 M sulfuric acid, 28 mM sodium phosphate, and 4 mM ammonium molybdate). The solution was incubated at 95 °C, and the absorbance was read at 695 nm (UV-2000 spectrophotometer) against a blank. BHT was used as a reference compound.

### 2.6. Antimicrobial Activity

#### Determination of MICs and MBCs

The determination of the minimum inhibitory concentrations (MICs) of *Lm*FV and Lin was based on the method previously reported by Ben akacha et al. [29]. In brief, the evaluation was performed using the broth microdilution method (96 microplates) with the addition of 10 µL of cell suspension and incubation overnight at 37 °C. The addition of thiazolyltetrazolium blue bromide (0.5 mg/mL) (Sigma-Aldrich, Taufkirchen, Germany) in all wells permitted visualization of the growth of microorganisms. MIC (%) was evaluated as the lowest concentration of the tested samples that inhibited the obvious growth of each tested bacterium. The concentrations of *Lm*FV and Lin were 30 mg/mL and 5 mg/mL, respectively.

The minimum bactericidal concentration (MBC) of *Lm*FV or Lin was determined by inoculating 10 µL of each well into MH stratum plates. The MBC was determined as the lowest concentration of *Lm*FV or Lin at which 99% of the bacteria were killed. The MIC and MBC tests were carried out three times.

### 2.7. Meat Sample Preparation and Conditioning

The raw beef was purchased from a local market (Sfax, Tunisia). Then, 15 g of meat was minced with a meat grinder. Within one hour of purchase, the samples were transported to the laboratory in polystyrene crates isolated on ice. For the packaging of raw ground beef, storage was at 4 °C. Five equal portions, as shown in Table 1, were placed separately in sterile plastic bags. The samples were stored at 4 °C for 14 days, and the quality parameters were checked on days 0, 3, 7, 10, and 14. 

#### 2.7.1. Microbiological Analysis

Microbiological counts of beef minced meat were determined following Ben Akacha et al. [29]. Aerobic plate counts (APCs) were estimated after incubation for 48 h at 30 °C using Plat Count Agar (PCA, Oxoid, Basingstoke, UK) medium. Total psychrotrophic counts (PTCs) are incubated at 7 °C for 10 days in PCA, Enterobacteria counts (ECs) are incubated at 37 °C for 24 h in red-violet bile glucose agar (VRBL, Oxoid, Basingstoke, UK), and *Salmonella* spp. were determined using Xylose-Lysine-Desoxycholate (XLD, Oxoid, Basingstoke, UK). After incubation at 37 °C for 24 h, to 10 g of sample, 90 mL of 0.1% sterilized buffered peptone water was added. After homogenization of the sample, 10-fold serial dilutions were prepared, and 1 mL of each dilution was plated on standard agar for plate counting standard agar. Microbiological counts were expressed as log CFU/g.

#### 2.7.2. Physicochemical Analysis (pH Analysis)

pH was assessed for the homogeneous mixtures of meat with distilled water using a proportion of 1:10 (*w*/*v*) [30]. A quantity of 50 mL of distilled water (pH 7.00) was used to homogenize 5 g of the sample before the mixture was filtered. A pH meter (Model: YK-21PH) was used to determine the pH of the filtrate at each sampling point.

#### 2.7.3. Physicochemical Analysis (Lipid Oxidation)

The extent of lipid oxidation in the different samples was evaluated by the determination of thiobarbituric-acid-reactive substances (TBARS) such as malondialdehyde (MDA). TBARS were assessed as reported by Eymard et al. [31]. Malonaldehyde equivalents in milligrams per kilogram of sample (mg/kg) were used to express the results.

#### 2.7.4. Physicochemical Analysis (Metmyoglobin (MetMb) Analysis)

Metamyoglobin (MetMb) was quantified as described by Wang et al. [32]. Briefly, 10 g of each meat sample was mixed with 50 mL of K3PO4 phosphate buffer (pH 6.8, 0.04 M) and centrifuged (3000× *g*, 30 min). After filtration, the absorbance of the filtrates was measured at 582, 525, 557, and 503 nm. The % MetMb was quantified using the formula provided by Wang et al. [32]:

MetMb% = [−2.51 (A572/A525) + 0.777 (A565/A525) + 0.8 (A545/A525) + 1.098] × 100(2)

### 2.8. Statistical Analysis

During refrigerated storage, measurements were taken at five-minute intervals, with experiments of five treatments used in a randomized complete block design. Three replications were also performed for each day of storage. A two-way analysis of variance (ANOVA) was performed for all variables, as well as for differences. The statistical significance of the differences between the mean values was investigated using Tukey’s test at a 5% significance level using the Statistical Package for the Social Sciences SPSS 21 (Ltd., Woking, UK). To group the five samples according to the parameters evaluated during the five days of storage, all variables were standardized before chemometric application.

Principal component analysis (PCA) and heat maps were used with XLSTAT software for Windows (v.2014.1.08, Addinsoft, New York, NY, USA) to separate the samples at 0, 3, 7, 10, and 14 days (n = 25). Pearson (n) was the PCA type. The dendrograms were constructed to provide a two-dimensional projection of the similarity or dissimilarity of the samples. A heat map was performed using Graphpad Prism 9.0 software.

## 3. Results and Discussion

### 3.1. Flavonoid Content of L. maritima Extract

**Figure 1:**
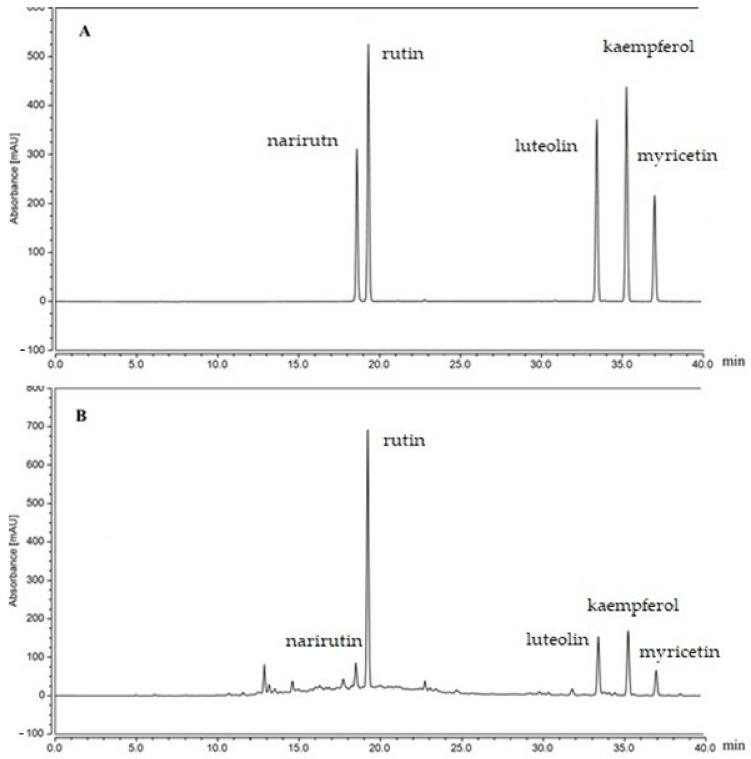
Chromatograms of flavonoid standards: (**A**) standard chromatograms; (**B**) flavonoid constituents of *L. maritima*.

The assay of flavonoid content in *L. maritima* was quantified by the external standard method (Figure 1). The HPLC results were represented as follows: narirutin (10 mg/g), rutin (84.9 mg/g), luteolin (27.3 mg/g), kaempferol (28.3 mg/g), and myricetin (8.4 mg/g).

### 3.2. Chemical Composition of L. maritima Extract after Derivatization

**Figure 2:**
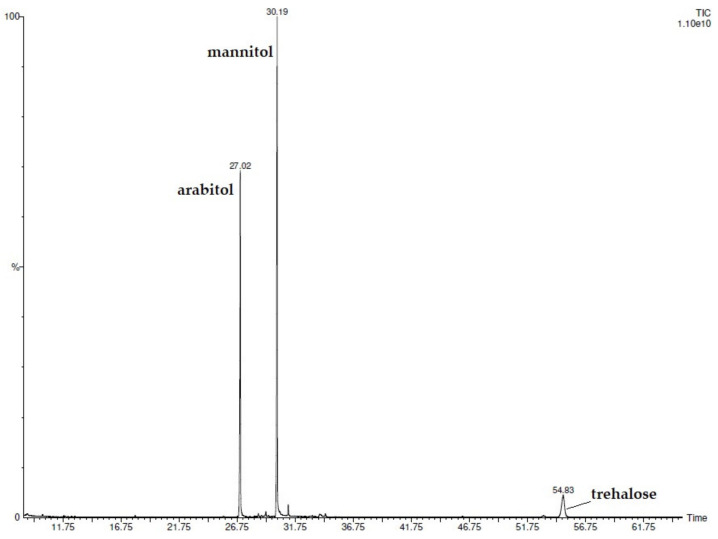
GC-FID chromatogram of *L. maritima* silylated ethanolic extract.

By the injection of the silylated extract, eighteen compounds belonging to different chemical classes (Table 2) were detected and identified (Figure 2). Sugar alcohols were the most abundant, with arabitol and mannitol reaching 36.1% and 49.6%, respectively. Among sugars, trehalose (10.4%) was the major component. Five fatty acids, including lauric, palmitic, linoleic, oleic, and stearic acids, were also determined, their percentage values ranging from 0.1 to 1.1%. Interesting was the presence of four terpenic compounds, although two of them (isoborneol and eugenol) were determined only in traces. The analyses were carried out in duplicate.

The presence of several identified compounds may be related to the antimicrobial activity shown by the extract. Indeed, sugars and sugar derivatives have numerous reported biological activities, including antimicrobial activity, and fatty acids are also known to possess antioxidant power [33,34].

To the best of our knowledge, this is the first work to report the following compositional profile of *L. maritima* ethanolic extract.

### 3.3. Antibacterial Activity

As illustrated in Table 3, *Lm*FV showed a potent antibacterial effect against *Listeria monocytogenes, Micrococcus luteus*, and *Salmonella enterica*, with an MIC value of 2.3 mg/mL for *Listeria monocytogenes*, thanks to the synergy of the myricetin and kaempferol flavones of the *L. maritima* extract. On the other hand, there was no inhibitory effect on *Pseudomonas aeruginosa* or *Bacillus cereus* (MIC >4 mg/mL), and this suggests the selective antibacterial effect of flavonoids on Gram-negative and -positive bacteria [35]. Flavonoids can exert several mechanisms of action against bacteria. They can interfere with lipid bilayers by inducing bacterial membrane disruption and inhibit several processes, such as biofilm formation, cell envelope synthesis, nucleic acid synthesis, the electron transport chain, and ATP synthesis [1,34]. Flavonoids (apigenin, chrysin, naringenin, and kaempferol) interfere with biofilm formation, while myricetin inhibits bacterial DNA replication [36,37]. On the other hand, Lin showed very potent antibacterial effects against the eight pathogenic strains, including those in which *Lm*FV is not very active, with MIC values ranging from 2.3 to 0.7 mg/mL. MBC values showed that Lin had a bacteriostatic effect on *Escherichia coli*, *Enterococcus faecalis*, and *Staphylococcus aureus* while having a bactericidal effect on *Pseudomonas aeruginosa*. Previous studies showed that Lin affected the structure of the cell envelope and could penetrate through the cell wall and destroy the cytoplasmic membrane; other results obtained by scanning electron microscopy showed that cell morphology was changed after treatment [20,38]. As a component of proton dynamics, PM is the potential difference between the inside and outside of biological cells, and it is closely related to ATP production [38].

The results presented in Table 2 show that the *Lm*FV sample isolated from *L. maritima* and the Lin sample are biologically active compounds that could potentially be used as food preservatives to improve the quality, shelf life, and safety of food.

### 3.4. Antioxidant Activity

In the present study, high antioxidant activity was recorded for the Lin sample. Indeed, the recorded results expressed by the IC_50_s were 5.64 and 24.73 µg/mL for the DPPH and phosphomolybdenum assays, respectively (Table 4). These results are in agreement with those of Meot-Duros et al. [39], who examined the DPPH scavenging activities of 21 authentic compounds, including Lin (the DPPH scavenging activity of Lin was 50.3 mg trolox equivalent/mL).

Considering the chemical structures of electron-donating flavonoids, the DPPH radicals could depend on the number of hydroxyl substitutions in their skeleton structures [16,37], as shown in the table below; the IC_50_ values were 50.78 and 87.02 µg/mL in the DPPH and phosphomolybdenum assays, respectively, which values were greater than the IC_50_s of BHT (22.38 and 51.87 µg/mL, respectively), and thus less effective than the positive control used. Previous studies have shown the antioxidant efficacy of *L. maritima* methanolic extracts thanks to the multitude of phenolic components [16] (total phenolic content: 175 ± 2.66 mg GAE/g DW, total flavonoids: 35 ±2.88 mg QE/g DW); the extract proved to be an active radical scavenger in in vitro tests (DPPH, lipid peroxidation inhibition test).

### 3.5. Microbiological Analysis

**Figure 3:**
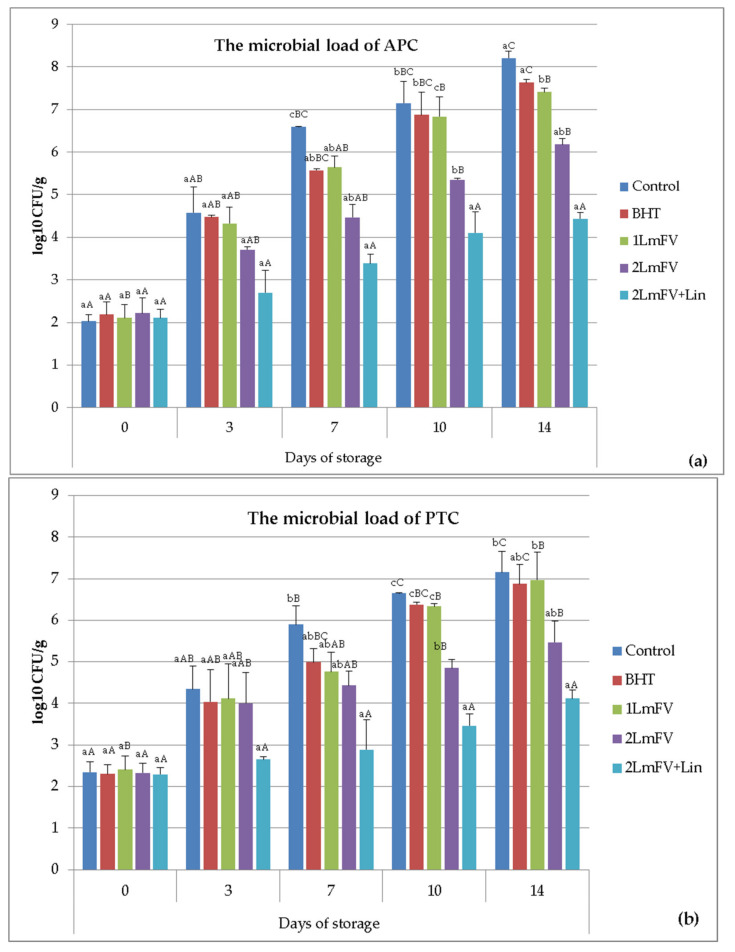
Effect of BHT, 1 *Lm*FV, 2 *Lm*FV, and 2 *Lm*FV+Lin on the microbial load of APC (**a**) and PTC (**b**) (log CFU/g) of raw minced meat samples stored at 4 °C for 14 days; means ± SEMs (n = 3). Values with a different letter (a–c) for the same storage day are significantly different (*p* < 0.05). Values with a different letter (A–C) for the same concentration are significantly different.

Figure 2 shows the comparison of the combined efficacy of *Lm*FV and Lin on the microbial stability of the raw ground beef samples stored at 4 °C. Overall, microbial counts showed significant differences (*p* < 0.05) over storage time. The APC counts (Figure 3a) of the five samples studied increased progressively over the storage period. The initial APC value was 2.10 log CFU/g, indicating the good quality of raw ground beef. It is necessary to mention that the PCA value of log 6.7 CFU/g represents the limit of the end of the microbiological life of raw ground beef according to the AFNOR V01-003 standard [40,41].

This value was exceeded on day 7 only for the control and on day 14 for the BHT, 1 *Lm*Fv, and 2 *Lm*FV samples, with values of 7.63, 7.41, and 6.18 log CFU/g, respectively. Interestingly, the APC counts recorded for sample 2 *Lm*FV+Lin remained below the detection limits until day 14 of storage.

Feng Xu et al. [42] revealed that flavonoids from *Sedum aizoon* L. rapidly reduced bacterial cell density and caused lysis of *Lactobacillus planetarum* in fresh pork meat by two antibacterial mechanisms: either by damaging the physical integrity of the cells or by increasing the electrical conductivity. It has also been shown that relatively small changes in the structural integrity of cell membranes can have a detrimental effect on cell metabolism and lead to cell death [43]. Sukhov et al. [44] suggested the existence of a possible direct relationship between antiradical activity and antimicrobial response which is expressed in a possible violation of the physiological redox signaling of microbial cells by the antiradical flavonoids. 

Therefore, other studies have shown that the addition of 0.5–2% green tea leaves and minerals is beneficial. Previously, these by-products were reported to reduce *Clostridium perfringens* levels in cooked ground beef, chicken, and pork during chilling abuse [45,46]. Although no information is available on the combination of flavonoids and Lin, this is a new combination that deserves to be investigated.

As illustrated in Figure 3b, the PTC numbers were significantly reduced in the 2 *Lm*FV+Lin treatments compared to the other treatments during the 14 days of refrigerated storage. The PTC of 2 *Lm*FV+Lin was inferior to that of 2 *Lm*FV alone (4.11 vs. 5.46 log CFU/g). In fact, according to the obtained results and the PTC values for the sample with 2 *Lm*FV + Lin, this treatment prolonged the shelf life of beef mince by about 10 days. Similar results were highlighted by Martnez et al. [47], who observed psychotropic count values between 6.10 and 5.06 log CFU/g for control and 1% black pepper (characterized by the presence of Lin) at days 8 and 9 of storage, respectively.

Until now, no data on the antimicrobial effects of flavonoids and Lin on beef minced meat have been reported.

For enterobacteria, counts were maintained below the threshold (2 log CFU/g) for meat treated with 4.6% *Lm*FV and 0.46% Lin during storage. All samples of meat treated with *Lm*FV had low counts (*p* < 0.05) compared to the control (Table 5).

Compared to the treatment with *Lm*FV alone, we observed a decrease in the growth of Enterobacteriaceae bacteria after the addition of Lin. Several studies have shown that Lin has a specific mechanism of action against foodborne pathogens by disrupting the normal morphology of the cell [20,27]. The release of nucleic acids as well as the decrease in membrane potential proved that the integrity of the membranes of the pathogens was destroyed [21,38]. On the other hand, other studies have shown that the treatment of beef meat with aqueous extract of *L. maritima* was able to inhibit the growth of Enterobacteriaceae and preserve its nutritional quality [39].

*Salmonella* spp. are pathogens that belong to the Enterobacteriaceae family [48]. According to the results presented in Table 4, *Salmonella* was not detected in any sample until the 7th day of storage for the control samples and BHT. On day 14, the number of strains increased slightly. Nevertheless, it can be observed that at the end of the storage period, all samples treated with *Lm*FV showed lower amounts than those detected in the untreated and BHT groups, with improved results in the treatment with Lin.

Overall, the application of *Lm*FV enriched with Lin as an active natural preservative formulation is a promising alternative for extending the storage period of ground meat and meat products by minimizing the growth rate of several bacteria.

### 3.6. Physicochemical Analyses

#### 3.6.1. pH Analysis

The pH values of the ground meat samples are shown in Table 6. The initial pHs were between 5.23 and 5.18. The pH values increased on the third day of storage and reached a maximum of 6.68 for the control sample. All samples continued to increase exponentially, except for the sample treated with 4.6% of *Lm*FV and 0.46% of Lin.

Incorporation of flavonoid fraction from *L. maritima* alone was ineffective against pH increase, which is in agreement with the results of Bozkurt et al. [49] and Brannan et Mah [50], who reported that flavonoids from green tea and seed oil were not effective in treating pH increase in pork and turkey meat. The pHs of the 2 *Lm*FV+Lin samples were significantly lower at the end of storage compared to those of the other samples.

Ben Akacha et al. [29] and Ben Hsouna et al. [39] also suggested that the incorporation of *L. maritima* essential oil, which contains Lin (22.43%), and aqueous extract from *L. maritima* in beef mince significantly reduced pH. These results show that the synergistic effect between Lin and *Lm*FV caused a decrease in pH, contributed to a reduction in the numbers of spoilage microorganisms, accelerated the reduction of nitrite to nitric oxide, affected the flavor of the product, and improved the binding capacity of the meat and its firmness [39], thus contributing to the safety of the product.

#### 3.6.2. Evaluation of Lipid Oxidation

The alteration of the flavor of meat and meat products is one of the unfavorable impacts of lipid oxidation, which lowers consumer approval of these products. A quantity of 0.2–0.6 mg MDA/kg TBARS caused an oxidized flavor in beef and reduced the acceptability of the meat as assessed by panelists, according to Grotta et al. [51]. A limit of 2 mg MDA/kg beef was reported to be acceptable by Campo et al. [19].

**Figure 4:**
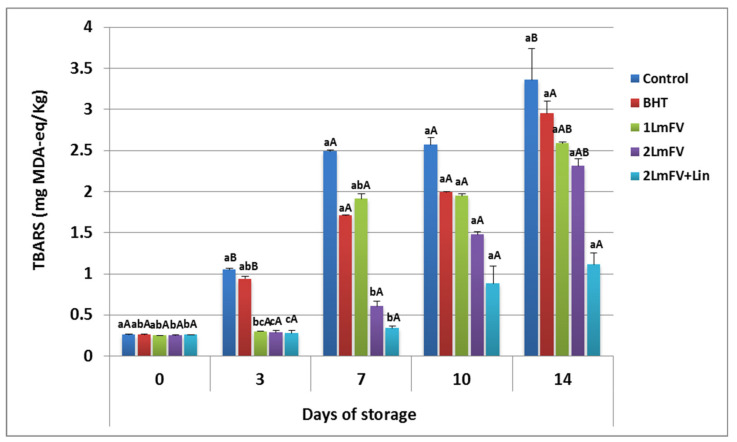
TBARS (mg MDA-eq/Kg) values for raw minced beef stored at 4 °C for 14 days; means ± SEMs (n = 3). Values with a different letter (a–c) for the same storage day are significantly different (*p* < 0.05). Values with a different letter (A,B) for the same concentration are significantly different.

The bovine meat samples were also evaluated for TBARS levels. These substances are used as crucial indicators for the evaluation of oxidation by-products, such as aldehydes [20]. They appear mainly as a result of lipid oxidation of polyunsaturated fatty acids [19]. TBARS levels increased significantly (*p <* 0.05) in all samples except the 2 *Lm*FV+Lin sample, as shown in Figure 4.

It is important to highlight that the activity of pro-oxidant microbial and endogenous enzymes, as well as the release of heme iron from myoglobin, should lead to a large increase in TBARS levels during storage [52]. At day 0, TBARS levels were similar (*p* > 0.05) in all meat samples. However, at the end of storage, TBARS levels in the untreated and BHT- or 1 *Lm*FV-treated meat samples were above the limit levels (3.36, 2.95, and 2.59 mg MDA, respectively). In contrast, TBARS levels were only 1.11 mg MDA equivalence per kilogram of meat treated with *Lm*FV (4.6%) combined with Lin (0.46%), which is below the acceptable level of TBARS (2 mg MDA eq/Kg of meat). Previous studies have shown that flavonoids present in grape seed extract at 0.1% (*w*/*w*) reduced TBRAS and hexanal in beef, chicken, turkey, and raw and cooked meats and caused a minor increase in red color retention [53,54].

From these results, it could be seen that the addition of Lin to *Lm*FV was the most effective treatment in retarding increase in TBARS values during storage. Therefore, this formulation may have reduced the oxidation of beef by amplifying the activity of free fatty acids.

#### 3.6.3. Evaluation of Protein Oxidation

**Figure 5:**
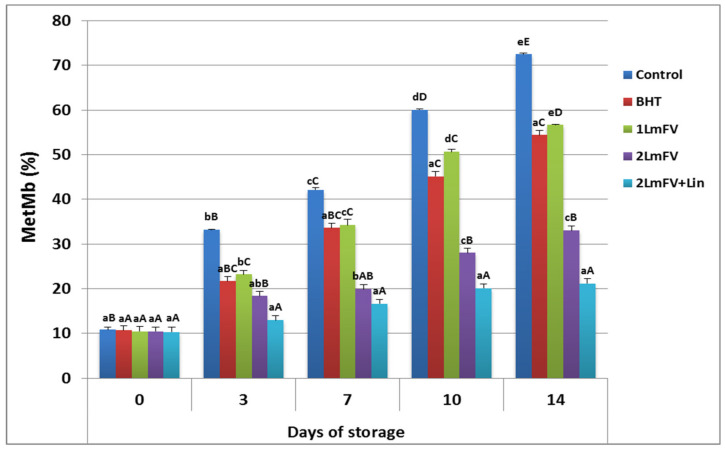
Effect of BHT, 1 *Lm*FV, 2 *Lm*FV, and 2 *Lm*FV+Lin on (a) MetMb (%) of raw minced beef stored at 4 °C for 14 days; means ± SEMs (n = 3). Values with a different letter (a–e) for the same storage day are significantly different (*p* < 0.05). Values with a different letter (A–E) for the same concentration are significantly different.

Figure 4 illustrates the rates of surface myoglobin formation in fresh beef during storage at 4 °C. The chemical state of myoglobin determines the color of the meat. In fact, oxidation of myoglobin to MetMb on the meat surface is frequently attributed to this unfavorable meat discoloration during storage [18,32]. The percentage of metmyoglobin increased rapidly during the first seven days of storage and reached values above 42.15% in the control sample, while for the samples treated with *Lm*FV the percentage of metmyoglobin ranged from 34.23 (1 *Lm*FV) to 19.92 (2 *Lm*FV). Consumer rejection having occurred at 40% MetMb in meat products [55], this limiting value occurred approximately after 14 days of storage for both BHT- and 1LmFV-treated samples (Figure 5). While this maximum MetMb value was not reached until the end of storage for the samples treated with 2 *Lm*FV and Lin (21.22%), the inhibition of protein oxidation in the sample treated with *Lm*FV+Lin after 14 days of storage, in terms of percentage of MetMb, was in agreement with the positive and synergistic effect between *Lm*FV and Lin that extends the shelf life of beef mince by about 7 days. In considering that the free radical scavengers of *Lm*FV and Lin could inhibit the formation of MetMb, our results can be explained by the potent antioxidant properties of the compositions present in the flavonoid fractions of *L. maritima* and the Lin. This is consistent with the results of Ben Hsouna et al. [39], who incorporated an aqueous extract of *L. maritima* into beef minced meat.

These extracts are high in flavone compounds (87.44 mg EQ/g) to prevent MetMb oxidation and preserve the red color of meat for up to 14 days. The ameliorative effect of the incorporation of Lin with *Lm*FV was evident during the first days of storage, and this formulation deserves to be explored as a natural preservative for minced meat.

### 3.7. Chemometric Analysis

To detect hidden patterns and similarities between the different samples according to the treatments used, the microbiological and physicochemical results over time were studied. The obtained data set was investigated using chemometric analysis, including the principal component analysis (PCA), the hierarchical cluster analysis (HCA), and the englobed in heat maps.

#### 3.7.1. Principal Component Analysis (PCA)

**Figure 6:**
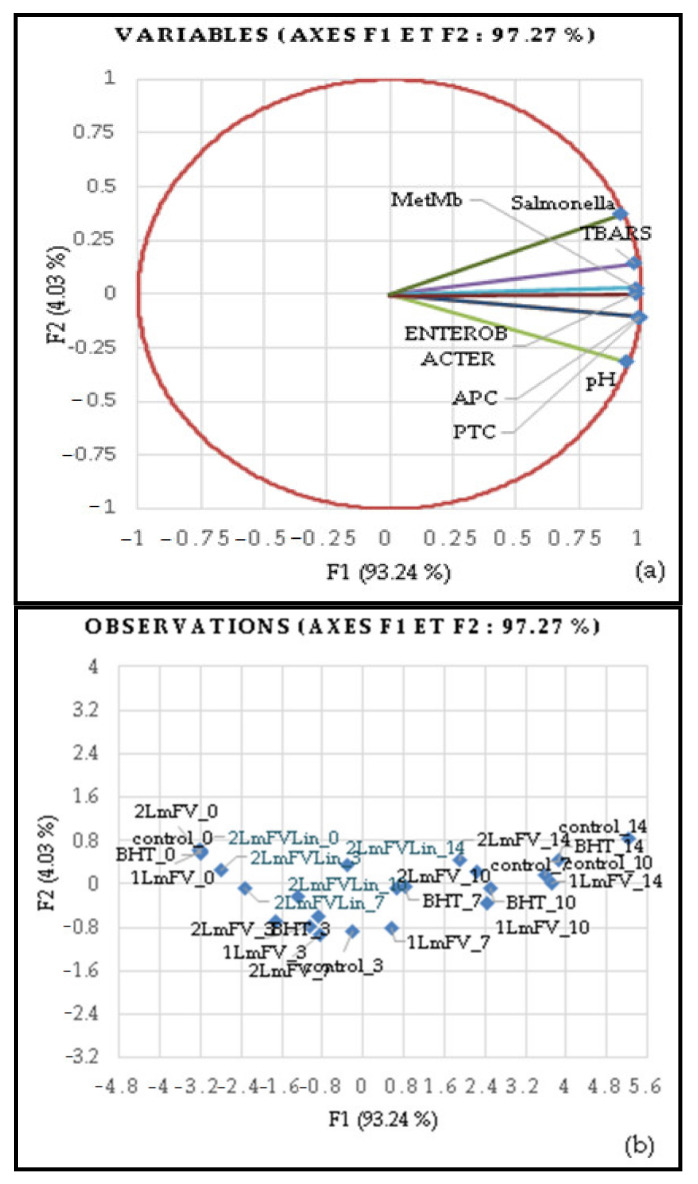
Loading plots of the two principal components (F1, F2) based on all samples (n = 25) (**a**) and on physicochemical and microbial growth of meat samples over 14 days of storage (**b**).

PCA was applied to the multivariate statistical analysis of the five samples that varied in quality and shelf life (0, 3, 7, 10, and 14 days). This analysis provided a view of the impact of treatments on the quality of lipid and protein oxidation and microbial growth in minced beef meat. The quality characteristics of the PCA were also correlated with the storage period (Figure 6a). This result accounted for 97.27% of the total variability in the data. In PCA 1, the horizontal axis is the primary axis and explains 93.24% of the variability, while PCA 2 is the secondary axis and explains 4.03 % of the total variability. The movement of samples (dots) from left to right in the PCA plot indicates continuous changes in chemical and microbiological properties during the storage period (Figure 6b).

With increasing storage time, samples treated with 4.6% of *Lm*FV and 0.46% of Lin exhibited the highest results, these substances acting as natural preservatives of meat quality and inhibiting the movement of scores to the right side of the graph, in contrast to the other treatments, including those with *Lm*FV alone. The antioxidant capacity of *L. maritima* flavonoid extracts was enhanced by the addition of Lin and was associated with improved oxidative stability and microbial contamination inhibition. Our findings are consistent with previous research that found a link between protein and lipid oxidation and an increase in bacterial load. Thus, Ben Hsouna et al. [39] demonstrated that *L. maritima* aqueous extracts can maintain the nutritional quality of meat over time. Based on microbiological, physicochemical, and sonority results, Ben Akacha et al. [29] demonstrated that the incorporation of essential oils from *L. maritima* significantly inhibits deterioration in the quality of minced meat.

It is worth noting that the improvement in the biopreservative effect of *Lm*FV by Lin appeared on the third day of storage in ground beef and slowed down lipid and protein oxidation, resulting in the preservation of high-quality meat until the end of the refrigerated shelf life.

#### 3.7.2. Hierarchical Cluster Analysis (HCA)

**Figure 7:**
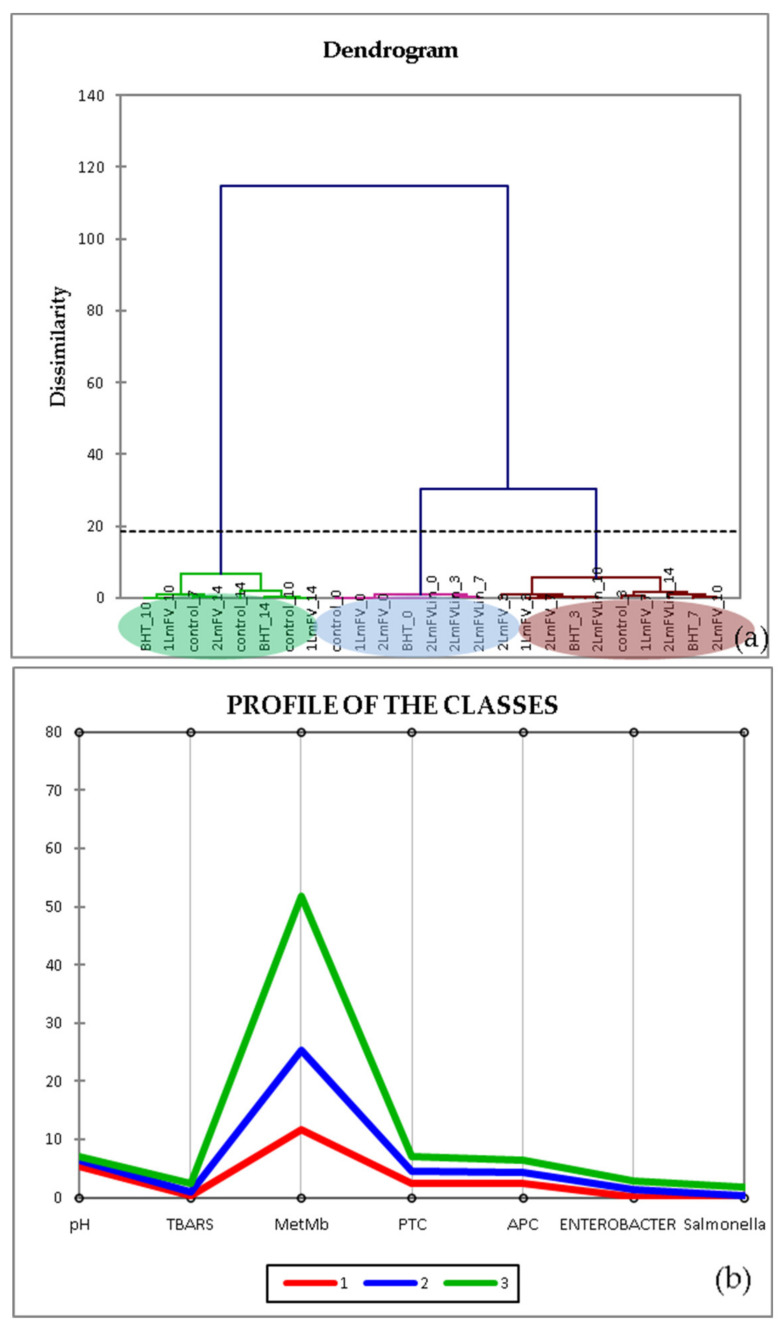
Agglomerative hierarchical cluster analysis (HCA) (**a**) and profile of classes (**b**) of physicochemical characteristics and microbial counts of different samples at each storage time: 0, 3, 7, and 14 days.

HCA is a clustering technique that is used to investigate the organizational pattern of a sample. In addition, illustrating the hierarchy helps to identify similarities and differences within and between groups [56]. This method was presented using the Euclidean distance matrix squared to obtain additional information on a probable classification of sample mixtures based on their contents and activities. The results of a HCA are usually illustrated in a dendrogram (Figure 7), a graph that shows the arrangement of samples and their relationships in tree form. Indeed, the generated dendrogram (Figure 7a) indicates the presence of three groups (1, (BHT_0-1 *Lm*FV_14), 2 (control_0-2 *Lm*FV_3), and 3 (1 *Lm*FV_7-2 *Lm*FV_10)), which corresponds to the PCA. The group marked in red presents, according to the class profile (Figure 6b), the best results for the inhibition of bacterial growth and oxidation processes over time. The sample 1 *Lm*FV+Lin_14 belongs to this group, which means that the strategy followed to improve the biopreservative effect of *Lm*FV was validated by this chemometric test.

#### 3.7.3. Heat Maps

A heat map is a two-dimensional tabular representation of data with a range of values represented by different colors, which provides a visual summary of information and helps to understand complex data sets.

**Figure 8:**
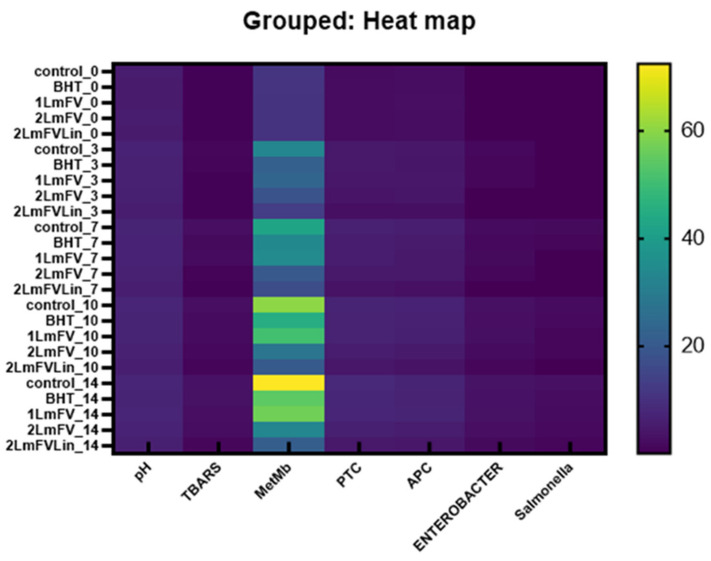
Heat maps of physicochemical properties and microbial growth for different samples during the refrigerated storage.

Figure 8 represents the correlation of the different samples according to the different parameters tested. The degree of correlation was between 20 and 60 with a positive sign, which means that all the factors influenced each other positively. This was confirmed by Zhao et al. [57]. According to these authors, a higher level of protein oxidation correlates with a higher level of TBARS. This results in a strong correlation between lipid and protein oxidation [40,57]. It has been commonly accepted that food-related oxidative processes, including lipid oxidation and enzymatic reactions, which use oxygen as a catalyst, are related to protein oxidation [52]. In addition, high microbial loads and protein oxidation parameters were associated with each other, which implied a change in the quality of the meat.

It is clear that chemometric instruments are widely used methods to assess the authenticity and quality of meat based on its oxidative stability and its effects during storage.

## 4. Conclusions

The findings of this study revealed that the fraction of *L. maritima*, as well as Lin, had significant antibacterial and antioxidant activity, and these were then used in combination to achieve an interesting biopreservative effect on raw ground beef during refrigerated storage at 4 °C. The formulation (4.6% *Lm*FV + 0.46% Lin) delayed the proliferation of spoilage microorganisms and limited lipid oxidation and MetMb accumulation, which consequently extended the shelf life by about 10 days. In conclusion, this formulation can be considered a solid and promising tool for future application as a safe method for the preservation of meat products.

## Figures and Tables

**Table 1:** Experimental conditions for sample preparation.

	Acronym	Experimental Conditions
Lot 1	Control	Untreated control
Lot 2	BHT	Processed with 0.01% BHT
Lot 3	1*Lm*FV	Processed with *Lm*FV at 2.3% (*v*/*w*)
Lot 4	2*Lm*FV	Processed with *Lm*FV at 4.6% (*v*/*w*)
Lot 5	2*Lm*FV+Lin	Processed with *Lm*FV at 4.6% and Lin at 0.46% (*v*/*w*)

**Table 2:** Chemical composition (percentage mean values) of *L. maritima* ethanolic extract after derivatization.

N°	COMPONENTS	*Lm*E. (%) ^1^
SUGAR ALCOHOLS		
1	Ribitol	0.1
2	Myo-inositol	0.1
3	Phytol	0.1
4	Glycerol	0.2
5	Arabitol	36.1
6	Mannitol	49.6
SUGARS		
7	Turanose	0.3
8	Mannobiose	0.7
9	Trehalose	10.4
FATTY ACIDS		
10	Lauric	0.1
11	Palmitic	1.1
12	Linoleic	0.3
13	Oleic	0.1
14	Stearic	0.4
TERPENES		
15	Isoborneol	tr
16	Farnesol	0.1
17	Neophytadiene	0.3
18	Eugenol	tr

^1^ Percentage values of the components of *L. maritima* ethanolic extract after derivatization. tr: traces (mean value < 0.1%).

**Table 3:** Antibacterial activity of *Lm*FV expressed as minimum inhibitory concentrations (MICs) and minimum bactericidal concentrations (MBCs).

Bacterial Strains	*Lm*FV (mg/mL)		Lin (mg/mL)			
	MIC	MBC	MIC	MBC	MBC/MIC	Antibacterial Activity
Gram-positive						
*Bacillus cereus* ATCC 14579	4.8 ± 1.05	>30	0.7 ± 0.55	>5	-	-
*Staphylococcus aureus* ATCC 25923	2.7 ± 0.95	>30	0.23 ± 0.08	5	21	Bacteriostatic
*Enterococcus faecalis* ATCC 29212	3.7 ± 0.00	>30	0.23 ± 0.08	2.5	10	Bacteriostatic
*Micrococcus luteus* ATCC 1880	2.7 ± 0.95	>30	0.23 ± 0.08	>5	-	-
*Listeria monocytogenes* ATCC 1911	2.3 ± 0.41	>30	0.46 ± 0.15	>5	-	-
Gram-negative						
*Pseudomonas aeruginosa* ATCC 9027	5.8 ± 0.12	>30	0.46 ± 0.15	1.2	2	Bactericidal
*Escherichia coli* ATCC 25922	3.7 ± 0.00	>30	0.38 ± 0.23	2.5	6	Bacteriostatic
*Salmonella enterica* ATCC 43972	2.75 ± 0.34	>30	0.46 ± 0.15	>5	-	-

Values are means ± SEMs (n = 3).

**Table 4:** IC_50_s of BHT, *Lm*FV, and Lin for different antioxidant systems.

Antioxidant Activity (IC_50_ (µg/mL))	BHT	*Lm*FV	Lin
DPPH radical scavenging activity	22.38 ± 0.34	50.78 ± 0.14	5.64 ± 0.25
Phosphomolybdenum assay	51.87 ± 0.46	87.02 ± 0.81	24.73 ± 0.27

Each value in the table represents the mean  ±  SEM (n  =  3).

**Table 5:** Enumeration of *Enterobacteriaceae* and Salmonella in various samples of raw ground beef stored at 4 °C for 14 days.

Samples	Days of Refrigerated Storage				
	0	3	7	10	14
*Enterobacteriaceae* counts					
Control	<1 ^aA^	1.47 ± 0.07 ^bAB^	1.80 ± 0.35 ^bB^	2.83 ± 0.14 ^dBC^	3.46 ± 0.47 ^aC^
BHT	<1 ^aA^	1.13 ± 0.13 ^bAB^	1.49 ± 0.45 ^abB^	2.62 ± 0.22 ^cBC^	3.26 ± 0.34 ^aC^
1*Lm*FV	<1 ^aA^	1.22 ± 0.07 ^bAB^	1.69 ± 0.28 ^bBC^	2.66 ± 0.55 ^cdCD^	3.16 ± 0.44 ^aD^
2*Lm*FV	<1 ^aA^	<1 ^aA^	1.08 ± 0.08 ^abAB^	2.03 ± 0.25 ^bBC^	2.53 ± 0.47 ^aC^
2*Lm*FV+Lin	<1 ^aA^	<1 ^aA^	<1 ^aA^	1.22 ± 0.02 ^aB^	1.72 ± 0.52 ^aB^
*Salmonella*					
Control	<1 ^aA^	<1 ^aA^	1.69 ± 0.01 ^cAB^	1.99 ± 0.06 ^bB^	2.93 ± 0.94 ^dB^
BHT	<1 ^aA^	<1 ^aA^	1.08 ± 0.12 ^bAB^	1.55 ± 0.62 ^bB^	2.11 ± 0.27 ^bB^
1*Lm*FV	<1 ^aA^	<1 ^aA^	<1 ^aA^	1.20 ± 0.13 ^bB^	1.91 ± 0.63 ^aB^
2*Lm*FV	<1 ^aA^	<1 ^aA^	<1 ^aA^	1.02 ± 0.02 ^bAB^	1.65 ± 0.77 ^bB^
2*Lm*FV+Lin	<1 ^aA^	<1 ^aA^	<1 ^aA^	<1 ^aA^	1.02 ± 0.29 ^aA^

Means ± SEMs (n = 3). Values with a different letter (a–d) for the same storage day are significantly different (*p* < 0.05). Values with a different letter (A–D) for the same concentration are significantly different.

**Table 6:** pH values of raw minced beef samples stored at 4 °C for 14 days.

Samples	Days of Refrigerated Storage				
	0	3	7	10	14
Control	5.21 ± 0.00 ^aA^	6.68 ± 0.02 ^bB^	6.94 ± 0.04 ^cC^	7.25 ± 0.05 ^dD^	7.46 ± 0.02 ^eE^
BHT	5.18 ± 0.00 ^aA^	6.34 ± 0.0 ^bB^	6.68 ± 0.08 ^bcBC^	6.94 ± 0.05 ^cC^	7.03 ± 0.02 ^cC^
1*Lm*FV	5.19 ± 0.02 ^aA^	6.37 ± 0.01 ^bB^	6.71 ± 0.03 ^cBC^	6.92 ± 0.01 ^dC^	7.25 ± 0.04 ^eD^
2*Lm*FV	5.23 ± 0.00 ^aA^	6.31 ± 0.1 ^abB^	6.59 ± 0.01 ^bB^	6.58 ± 0.02 ^bB^	6.69 ± 0.01 ^bB^
2*Lm*FV+Lin	5.19 ± 0.01 ^aA^	5.52 ± 0.02 ^abA^	5.82 ± 0.08 ^cA^	6.02 ± 0.02 ^cdA^	6.17 ± 0.02 ^dA^

Means ± SEMs (n = 3). Values with a different letter (a–e) for the same storage day are significantly different (*p* < 0.05). Values with a different letter (A–E) for the same concentration are significantly different.

## Data Availability

The data presented in this study are available privacy on request from Anis Ben Hsouna.
